# Atopische Dermatitis ist bei Erwachsenen mit Herpes zoster assoziiert – eine gepaarte Kohortenstudie auf Basis elektronischer Patientenakten in England

**DOI:** 10.1111/ddg.70329x

**Published:** 2026-09-08

**Authors:** Anna K. Schober, Sinéad M. Langan, Hywel C. Williams, Harriet Forbes, Julian Matthewman

**Affiliations:** ^1^ Department of Non‐Communicable Disease Epidemiology London School of Hygiene and Tropical Medicine Vereinigtes Königreich Großbritannien und Nordirland London Nordirland; ^2^ Centre of Evidence Based Dermatology Lifespan and Population Health, School of Medicine, University of Nottingham, Nottingham, Vereinigtes Königreich Großbritannien und Nordirland London Nordirland

**Keywords:** Atopische Dermatitis, atopisches Ekzem, elektronische Patientenakten, Ekzem, Gürtelrose, Herpes zoster, Kohortenstudie, Atopic dermatitis, atopic eczema, cohort study, eczema, electronic health records, herpes zoster, shingles

## Abstract

**Hintergrund und Ziele:**

Manche Therapien für atopische Dermatitis (AD) scheinen mit erhöhtem Risiko für Herpes zoster (HZ) einherzugehen. Es gibt auch begrenzte Evidenz für eine unabhängige Assoziation zwischen AD und HZ. Die Studie hatte das Ziel, die Assoziation zwischen AD und HZ im Vereinigten Königreich zu untersuchen und die Rolle von Behandlungen dabei zu erforschen.

**Patienten und Methoden:**

Wir führten eine gepaarte Kohortenstudie mit routinemäßig erfassten Daten der Primärversorgung aus der Datenbank Clinical Practice Research Datalink (CPRD) Aurum (1997–2023) des Vereinigten Königreichs durch und wandten dabei Cox‐Regressionsmodelle an. Alter, Verhaltensfaktoren, mehrere Begleitkrankheiten und verschiedene Therapien wurden als Kovariablen eingeschlossen.

**Ergebnisse:**

Patienten mit AD hatten eine adjustierte *Hazard Ratio* von 1,28 (95 %‐KI: 1,27–1,29) für HZ. Adjustierungen für verschiedene Definitionen der Exposition gegenüber oralen Kortikosteroiden sowie anderen herkömmlichen Immunsuppressiva führten zu minimalen Reduktionen bei der Assoziation. Das Risiko für HZ stieg mit dem Schweregrad der AD (aHR für leichte AD: 1,22 [95 %‐KI: 1,20–1,23]; aHR für mittelschwere AD: 1,28 [95 %‐KI: 1,27–1,30]; aHR für schwere AD: 2,33 [95 %‐KI: 2,22–2,45]).

**Schlussfolgerungen:**

Das Risiko für HZ ist bei Patienten mit AD unabhängig von der Komorbidität und der Verwendung oraler Kortikosteroide oder Immunsuppressiva erhöht. Dieses erhöhte Grundrisiko ist besonders relevant, da HZ auch eine bekannte Nebenwirkung neuerer Therapien wie Januskinase‐Inhibitoren (JAKi) ist. Diese Ergebnisse können für Impfrichtlinien relevant sein.

## EINLEITUNG

Atopische Dermatitis (AD), auch bekannt als atopisches Ekzem,^1^ ist eine chronisch‐entzündliche Hautkrankheit, die eine erhebliche Belastung für die Patienten und das Gesundheitssystem hinsichtlich der krankheitsbereinigten Lebensjahre darstellt.^2^, ^3^ Die Prävalenz ist zwar bei Kindern am höchsten, es besteht jedoch auch bei Erwachsenen eine bedeutende Krankheitslast. In Europa reicht die Prävalenz bei Erwachsenen von 2,2 % in Deutschland über 4,3 % im Vereinigten Königreich bis 8,1 % in Italien und wird insgesamt auf etwa 4,4 % geschätzt.^4^, ^5^, ^6^ Die Erkrankung kann sich sowohl chronisch als auch schubförmig durch starken Juckreiz und ekzematöse Hautveränderungen manifestieren.^7^

Bei therapieresistenter Erkrankung benötigen Patienten mit schwerer AD möglicherweise eine systemische Behandlung.^8^ Diese kann orale Kortikosteroide für akute Schübe und Immunsuppressiva wie Ciclosporin oder Methotrexat für die laufende Behandlung umfassen. In den letzten Jahren wurden mehrere Biologika sowie Behandlungen mit niedermolekularen Wirkstoffen entwickelt und als zusätzliche vielversprechende Therapieansätze zugelassen.^9^

In früheren Forschungsarbeiten zur AD wurden mehrere Assoziationen mit anderen Krankheiten ermittelt, einschließlich einer erhöhten Anfälligkeit gegenüber systemischen Infektionskrankheiten.^10^, ^11^ Laut einer aktuellen Studie^12^ gehören Hautinfektionen, einschließlich Herpes simplex, zu den unerwünschten Ereignissen, die am stärksten mit AD assoziiert sind. Allerdings wurde in dieser Studie die Assoziation mit Herpes zoster nicht untersucht.

Herpes zoster (HZ), allgemein bekannt als Gürtelrose, wird durch die Reaktivierung von latentem Varizella‐Zoster‐Virus (VZV) in kutanen Nerven verursacht und betrifft insbesondere ältere Erwachsene und immunsupprimierte Personen.^13^, ^14^ HZ manifestiert sich als abgegrenzter, schmerzhafter, unilateraler Ausschlag im entsprechenden Dermatom.^15^

Die mögliche Assoziation zwischen AD und HZ wurde vor allem im Zusammenhang mit AD‐Behandlungen diskutiert. Insbesondere systemische Kortikosteroide und Immunsuppressiva wurden mit erhöhtem Risiko für HZ in Verbindung gebracht.^16^, ^17^ Das Gleiche gilt für JAK‐Inhibitoren (JAKi), einer Subklasse neu entwickelter niedermolekularer therapeutischer Wirkstoffe.^8^ Allerdings ist es wahrscheinlich, dass Patienten mit AD aufgrund der veränderten Immunantwort der Haut und der früher beobachteten erhöhten Anfälligkeit für kutane und extrakutane Infektionen^10^, ^18^ unabhängig von ihrer Behandlung ein erhöhtes Risiko für HZ aufweisen.

Es gibt zwar einige Hinweise, die auf ein erhöhtes HZ‐Risiko bei Personen mit AD unabhängig von systemischen Behandlungen schließen lassen,^19^, ^20^, ^21^, ^22^ insgesamt ist die Evidenz hinsichtlich Stärke, Ausmaß und Generalisierbarkeit der Assoziation zwischen AD und HZ jedoch begrenzt. Eine große, im Vereinigten Königreich durchgeführte Kohortenstudie untersuchte insbesondere das „VZV‐Infektionsrisiko“, ohne jedoch Windpocken‐ oder HZ‐Diagnosen spezifisch zu betrachten.^19^ Andere Studien weisen mangelhafte Genauigkeit auf^20^, ^21^ oder basieren auf Krankenhausdaten^22^ und repräsentieren daher nicht die allgemeine Bevölkerung.

In Anbetracht der Impfstoffentwicklungen und breiter Einführung von Impf‐ und Präventionsprogrammen können weitere und genauere Untersuchungen der möglichen Assoziation angemessene neue Informationen zu Impfrichtlinien und stratifizierten Risikobewertungen für Personen mit AD liefern.

Diese Studie hat das Ziel, den Zusammenhang zwischen AD und HZ bei Erwachsenen anhand von Daten der Primärversorgung des Vereinigten Königreichs zu untersuchen und dabei gleichzeitig die komplexe Rolle systemischer immunmodulierender Behandlungen und der Exposition gegenüber oralen Kortikosteroiden zu berücksichtigen.

## PATIENTEN UND METHODEN

Dies ist eine gepaarte Kohortenstudie mit routinemäßig erfassten Daten der Primärversorgung aus der Datenbank Clinical Practice Research Datalink (CPRD) Aurum des Vereinigten Königreichs. CPRD Aurum ist eine große Forschungsdatenbank mit über 46 Millionen routinemäßig erfassten und anonymisierten elektronischen Patientenakten (ePA) der Primärversorgung in England. Sie ist repräsentativ für die englische Bevölkerung hinsichtlich geographischer Verteilung, sozialer Benachteiligung, Geschlecht und Alter.^23^

Personen mit AD in der Datenbank wurden anhand eines früher validierten Algorithmus identifiziert, der eine Kombination aus entsprechendem Diagnose‐Code und mindestens zwei AD‐bedingten Verschreibungen berücksichtigte.^24^ Das Indexdatum (Datum der Aufnahme in die Kohorte) wurde als letztes der folgenden Daten definiert:

– das Datum an dem die AD‐Definition erfüllt wurde;

– ein Jahr nach Registrierung bei einem Hausarzt;

– das Startdatum der Studie (1. April 1997); oder

– das Datum, and dem die Person 18 Jahre alt wurde.

Dies ergab eine Kohorte, in der sowohl inzidente als auch prävalente Fälle enthalten waren.^25^ Personen in der AD‐Kohorte wurden dann hinsichtlich des Alters (± 2 Jahre), Geschlechts und der Allgemeinpraxis mit bis zu fünf nicht exponierten Vergleichspersonen gepaart, das heißt mit Personen, die die AD‐Definition nicht erfüllten. Die Vergleichspersonen wurden bis zum selben Datum nachverfolgt wie die entsprechende exponierte Person.

Wenn Vergleichspersonen während der Nachkontrolle die AD‐Definition erfüllten, wurden sie zensiert und anschließend als exponierte Patienten erneut gepaart. Ansonsten wurden alle Personen am ersten der folgenden Daten zensiert:

– Diagnose des Studienendpunkts (HZ);

– Datum des Verlassens der Hausarztpraxis;

– Tod; oder

– Studienende (31. März 2023).

Beobachtungen wurden aus der Studie ausgeschlossen, wenn eine HZ‐Diagnose vor dem Indexdatum in den Akten dokumentiert war. Die Analyse war auf gepaarte Sätze beschränkt, die sowohl exponierte als auch nicht exponierte Personen enthielten. Der relevante Endpunkt war die erste dokumentierte Diagnose von HZ (siehe Code‐Liste, Online‐Supplement S1).

Mehrere Kovariablen wurden berücksichtigt und als mögliche Einflussfaktoren in die Analyse eingeschlossen. Dazu gehörten eine Reihe chronischer Begleitkrankheiten wie Asthma,^19^, ^26^, ^27^ allergische Rhinitis,^19^, ^26^, ^28^ COPD,^19^, ^28^, ^29^ koronare Herzkrankheit,^28^, ^30^ Morbus Crohn,^19^, ^31^, ^32^ Depression,^19^, ^28^, ^33^ Diabetes,^19^, ^28^, ^34^, ^35^ Herzinsuffizienz,^19^ arterielle Hypertonie^28^, ^34^ und Colitis ulcerosa.^19^, ^31^, ^32^ Eine Dokumentation dieser Begleitkrankheiten vor dem Indexdatum des Patienten wurde als positive Anamnese dieser Erkrankung betrachtet. Eine Diagnose von Adipositas, Rauchstatus oder Alkoholabusus innerhalb von 2 Jahren vor dem Indexdatum wurde als positiv für diese Kovariablen betrachtet. Quintile der Werte für den Index multipler Benachteiligungen (*Index of multiple deprivation*; IMD) wurden aus der IMD‐Datenbank extrahiert, um die soziale Benachteiligung zu bewerten.^36^ Informationen zur ethnischen Herkunft wurden den Beobachtungdaten von CPRD Aurum entnommen.^37^, ^38^

Der Status der Medikamentenexposition wurde anhand der CPRD Aurum Verschreibungsdaten definiert. Binäre zeitaktualisierte Variablen wurden erstellt, um zu bestimmen, ob die Patienten jemals oralen Kortikosteroiden und/oder systemischen Immunsuppressiva (Methotrexat, Azathioprin, Ciclosporin, Mycophenolat) ausgesetzt waren, definiert als „erste Exposition erfolgte innerhalb von 180 Tagen vor dem Indexdatum oder während der Nachkontrolle“. Um die Wirkungen oraler Kortikosteroide genauer zu untersuchen, berücksichtigten wir zudem Verschreibungen im Jahr vor dem Indexdatum und die Aktualität der letzten Verschreibung (< 30 Tage, 30–90 Tage, 90–180 Tage und > 180 Tage) als zeitaktualisierte Variablen.

Der AD‐Schweregrad wurde anhand der Behandlungsmuster eingeschätzt: Die AD der Patienten wurde als mittelschwer eingestuft, wenn sie Verschreibungen für potente topische Kortikosteroide oder Calcineurin‐Inhibitoren erhalten hatten. Bei Behandlung mit systemischen Immunsuppressiva oder einer dokumentierten Phototherapie wurde die AD als schwer eingestuft. Ansonsten wurde die AD der Patienten als leicht eingestuft. Der zugewiesene Schweregrad konnte zu einem höheren Grad fortschreiten, aber nicht zu einem geringeren Grad zurückfallen. Dieses Vorgehen stimmt mit früheren Studien überein. Aufgrund begrenzter Datenverfügbarkeit wurden Krankenhausakten nicht eingeschlossen.^12^ Alle verwendeten Code‐Listen sind im Repository einer früheren Studie zu finden.^12^

Zur Berechnung von *Hazard Ratios* (HRs) und den zugehörigen 95 %‐Konfidenzintervallen (KIs) wurden stratifizierte *Cox‐Proportional‐Hazards*‐Regressionsmodelle eingesetzt. Die Variablen der Fallpaarungen wurden implizit berücksichtigt. Der Kalenderzeitraum der Nachkontrolle wurde durch gepaarte Indexdaten einbezogen. Ein univariables stratifiziertes Modell wurde zunächst angepasst, um die Assoziation unter alleiniger Berücksichtigung der Faktoren der Paarungen zu untersuchen. Anschließend wurde das Modell zu einem multivariablen Modell ausgeweitet, um die verbleibenden Kovariablen einzuschließen. Danach wurden verschiedene Definitionen für Behandlungsexpositionen hinzugefügt, um deren Rolle bei der Assoziation zu bewerten. Um den Einfluss des Alters auf die Effektschätzer zu untersuchen, wurden die Beobachtungen in Altersbereiche von 10 Jahren aufgeteilt und es wurde eine Interaktionsgröße zwischen AD und Alter aufgenommen. Schoenfeld‐Residuen wurden bewertet, um die Zeitabhängigkeit zu beurteilen. Aufgrund des hohen Anteils fehlender Daten, wurden IMD‐Scores (13,6 % fehlende Daten) und ethnische Herkunft (24,6 % fehlende Daten) nicht als Kovariablen bei der statistischen Hauptanalyse berücksichtigt.

Anhand von Sensitivitätsanalysen wurde untersucht, wie die unterschiedlichen Einschlusskriterien die Effektschätzer beeinflussten. Dazu gehörten die Beschränkung der Kohorte auf inzidente AD‐Fälle (bei denen das Indexdatum dem Datum der AD‐Diagnose entsprach) und das Entfernen der Altersbeschränkung, um Kinder mit aufzunehmen. Bei einer weiteren Sensitivitätsanalyse wurden die Beobachtungen auf Personen mit mindestens einer von vier gebräuchlichen Aufzeichnungen im Jahr vor dem Indexdatum beschränkt (9N11.00 – Besuch in Praxis eines Hausarztes; 22A¨00 – Körpergewicht; 4….00 – Labortest; 246¨00 – O/E – Blutdruckmessung). Außerdem wurden die Daten hinsichtlich ethnischer Herkunft und IMD adjustiert.

## ERGEBNISSE

### Ausgangscharakteristika

Insgesamt wurden 14.081.774 Beobachtungen von 13.426.267 Patienten in die Studie eingeschlossen: 11.636.684 Beobachtungen von Teilnehmern ohne AD‐Exposition und 2.445.090 Beobachtungen von Teilnehmern mit AD‐Exposition (1.382.577 inzidente Fälle und 1.062.513 prävalente Fälle). Insgesamt wurden 84.341.513 Personenjahre gefährdeter Populationen (15.729.178 Jahre mit Exposition und 68.612.336 Jahre ohne Exposition) beobachtet. Es kam zu insgesamt 329.821 HZ‐Ereignissen: 76.990 bei Patienten mit AD und 252.831 in der gepaarten Vergleichsgruppe. Eine umfassende Übersicht der Charakteristika der Teilnehmer ist in Tabelle 1 gezeigt.

**TABELLE 1:** Ausgangscharakteristika der Patienten (n, %) stratifiziert nach AD‐Expositionsstatus. Da Patienten Personenzeit zu beiden Gruppen beitragen konnten, spiegelt die Summe der Beobachtungen nicht die Gesamtzahl der Teilnehmer wider.

	Keine AD (n = 11.636.684)		AD (n = 2.445.090)	
	*n*	*%*	*n*	*%*
Zoster‐Ereignisse	252.831	(2,2 %)	76.990	(3,1 %)
Alter (Mittelwert [SD])	40,7	(20,5)	41,4	(20,9)
Geschlecht				
W	6.750.742	(58,0 %)	1.422.616	(58,2 %)
M	4.885.934	(42,0 %)	1.022.466	(41,8 %)
I	8	(< 0,1 %)	8	(< 0,1 %)
IMD^2^				
1 (geringste Benachteiligung)	2.033.670	(17,5 %)	435.207	(17,8 %)
2	2.041.860	(17,5 %)	430.571	(17,6 %)
3	1.984.581	(17,1 %)	411.505	(16,8 %)
4	2.054.895	(17,7 %)	427.836	(17,5 %)
5 (höchste Benachteiligung)	1.935.259	(16,6 %)	404.905	(16,6 %)
Daten fehlen	1.586.419	(13,6 %)	335.066	(13,7 %)
Ethnische Herkunft				
Weiß	6.024.957	(51,8 %)	1.301.908	(53,2 %)
Südasiatisch	554.860	(4,8 %)	138.197	(5,7 %)
Schwarz	279.368	(2,4 %)	55.606	(2,3 %)
Sonstige	1.496.796	(12,9 %)	365.280	(14,9 %)
Gemischt	91.613	(0,8 %)	17.853	(0,7 %)
Keine Angabe	278.563	(2,4 %)	51.873	(2,1 %)
Daten fehlen	2.910.527	(25,0 %)	514.373	(21,0 %)
Alkoholabusus	42.954	(0,4 %)	13.434	(0,5 %)
Zigarettenrauchen	2.121.192	(18,2 %)	519.926	(21,3 %)
Adipositas	439.161	(3,8 %)	137.096	(5,6 %)
***Positive Anamnese***				
Asthma	1.485.154	(12,8 %)	602.427	(24,6 %)
Allergische Rhinitis	836.455	(7,2 %)	358.844	(14,7 %)
Herzkrankheit	660.471	(5,7 %)	182.288	(7,5 %)
COPD	180.763	(1,6 %)	58.171	(2,4 %)
Morbus Crohn	23.439	(0,2 %)	8.976	(0,4 %)
Depression	1.497.428	(12,9 %)	440.430	(18,0 %)
Diabetes	489.087	(4,2 %)	129.717	(5,3 %)
Herzinsuffizienz	113.011	(1,0 %)	32.526	(1,3 %)
Arterielle Hypertonie	1.414.772	(12,2 %)	361.940	(14,8 %)
Colitis ulcerosa	39.823	(0,3 %)	13.517	(0,6 %)

*Abk*.: AD, atopische Dermatitis; COPD, chronisch obstruktive Lungenerkrankung; HZ, Herpes zoster; IMD, Index multipler sozialer Benachteiligungen; SD, Standardabweichung

^1^ p‐Werte wurden für kategorische Variablen aus χ^2^‐Tests und für kontinuierliche Variablen aus t‐Tests abgeleitet.

^2^ Quintile des Index multipler Deprivation.

### Inzidenz

Insgesamt betrug die rohe Inzidenzrate für HZ in der gesamten Kohorte 3,91 (95 %‐KI: 3,90–3,92) je 1.000 Personenjahre. Die rohen Inzidenzraten für HZ stiegen mit dem Alter kontinuierlich an; von 1,58 Ereignissen je 1.000 Personenjahre bei Patienten im Alter < 30 Jahre auf 8,85 Ereignisse je 1.000 Personenjahre bei Patienten im Alter ≥ 80 Jahre. In der nicht exponierten Gruppe war die Rate mit 3,68 Ereignissen je 1.000 Personenjahre (95 %‐KI: 3,67–3,70) etwas geringer als in der exponierten Gruppe mit 4,90 (95 %‐KI: 4,86–4,93) Ereignissen je 1.000 Personenjahre.

### Regressionsmodelle

In den stratifizierten Modellen, in denen die Faktoren der Paarungen berücksichtigt wurden, hatten Patienten mit AD ein 1,33‐fach höheres Risiko, an HZ zu erkranken, als ihre gepaarten Vergleichspersonen ohne AD (95 %‐KI: 1,31–1,34) (Tabelle 2).

**TABELLE 2:** Überblick über die Ergebnisse der univariablen und multivariablen Cox‐Regressionsmodelle: *Hazard Ratios* und 95 %‐KIs.

	HR	95 %‐KI
***Univariables Modell*** ^1^		
AD	1,33	(1,31–1,34)
***Multivariables Modell*** ^1^, ^2^		
AD	1,28	(1,27–1,29)
***Multivariables Modell*** ^1^, ^2^ ***+ unterschiedliche Definitionen für Exposition gegenüber oralen Kortikosteroiden***		
Orale Kortikosteroide bei Studienbeginn^3^		
AD	1,27	(1,26–1,28)
Orale Kortikosteroide (zeitaktualisiert)		
AD	1,24	(1,23–1,25)
Orale Kortikosteroide Aktualität (zeitaktualisiert^4^)		
AD	1,25	(1,24–1,26)
Kortikosteroide (Aktualität)		
< 30 Tage	2,00	(1,95–2,05)
30–90 Tage	1,62	(1,57–1,67)
90–180 Tage	1,47	(1,42–1,52)
> 180 Tage	1,27	(1,26–1,29)
***Multivariables Modell*** ^1^, ^2^ ***+ Immunsuppressiva (zeitaktualisiert)***		
AD	1,27	(1,26–1,28)
Systemische Immunsuppressiva	2,06	(2,01–2,12)

*Abk*.: AD, atopische Dermatitis; KI, Konfidenzintervall; HR, *Hazard Ratio*

^1^ Implizit korrigiert für Alter, Geschlecht, Allgemeinpraxis und Kalenderzeitraum durch Fallpaarungen am Indexdatum.

^2^ Adjustiert für Asthma, allergische Rhinitis, COPD, koronare Herzkrankheit, Morbus Crohn, Depression, Diabetes, Herzinsuffizienz, arterielle Hypertonie, Colitis ulcerosa, Alter bei Studienbeginn, Adipositas, Zigarettenrauchen und Alkoholabusus.

^3^ Aufzeichnung der Kortikosteroid‐Verschreibung im Jahr vor dem Indexdatum.

^4^ Zeit seit letzter erfasster Kortikosteroid‐Verschreibung.

Anschließend wurde das Modell zusätzlich für folgende Faktoren adjustiert: Asthma, allergische Rhinitis, COPD, koronare Herzkrankheit, arterielle Hypertonie, Herzinsuffizienz, Colitis ulcerosa, Morbus Crohn, Diabetes, Depression, Alkoholabusus, Zigarettenrauchen und Adipositas. Diese Adjustierungen verringerten die Effektschätzer leicht auf eine adjustierte HR (aHR) von 1,28 (95 %‐KI: 1,27–1,29).

Im Vergleich zu Patienten ohne AD stieg das Risiko für HZ mit steigendem AD‐Schweregrad an (leichte AD: aHR 1,22 [95 %‐KI: 1,20–1,23]; mittelschwere AD: aHR 1,28 [95 %‐KI: 1,27–1,30]; schwere AD: aHR 2,33 [95 %‐KI: 2,22–2,45]).

### Effekt von Behandlungen

Während der Nachkontrolle wurden 21,1 % der Patienten mit AD orale Kortikosteroide verschrieben, im Vergleich zu 9,7 % der Patienten ohne AD. Potente topische Kortikosteroide wurden 49,8 % der Patienten mit AD und 10,0 % der Patienten ohne AD verschrieben, während systemische Immunsuppressiva 1,9 % der Patienten mit AD und 0,9 % der Patienten ohne AD verschreiben wurden.

Die Adjustierung für orale Kortikosteroide führte nur zu einer geringen Verringerung der adjustierten Effektschätzer (Tabelle 2). Dies wurde beobachtet, wenn die Exposition gegenüber oralen Kortikosteroiden vor dem Indexdatum (aHR: 1,27; 95 %‐KI: 1,26–1,28), als zeitabhängige Kovariable (aHR: 1,24; 95 %‐KI: 1,23–1,25) oder gemäß der zeitaktualisierten letzten Verschreibung (aHR: 1,25; 95 %‐KI: 1,24–1,26) bewertet wurde. Die individuelle Adjustierung für zeitaktualisierte systemische Immunsuppressiva führte zu einer aHR von 1,27 (95 %‐KI: 1,26–1,28), während die Adjustierung für topische Kortikosteroide eine aHR von 1,17 (95 %‐KI: 1,16–1,18) ergab.

### Interaktion mit dem Alter

**ABBILDUNG 1:**
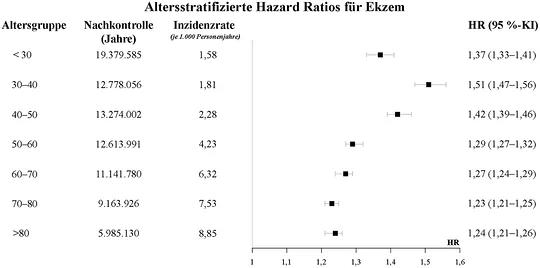
*Forest‐Plot* mit den *Hazard Ratios* (HRs) und 95 %‐KIs für die Assoziation zwischen AD und Herpes zoster stratifiziert nach Altersgruppe, einschließlich einer Übersicht über Dauer der Nachkontrolle und Inzidenzen für jede Gruppe.

Es gab Hinweise auf eine Effektmodifikation durch das Alter bei der Assoziation zwischen AD und HZ (*Likelihood*‐Quotienten‐Test: p < 0,001), wobei höhere HR für AD bei Personen unter 50 Jahren im Vergleich zu Gruppen mit höherem Lebensalter beobachtet wurden (Abbildung 1, Tabelle 3). Patienten im Alter von 30–40 Jahren wiesen die höchsten HRs auf (HR: 1,51; 95 %‐KI: 1,47–1,56), gefolgt von denen im Alter von 40–50 Jahren (HR: 1,42; 95 %‐KI: 1,39–1,46), während sie in den Altersgruppen über 50 Jahre durchgehend niedriger waren.

**TABELLE 3:** Inzidenzraten für Herpes zoster je 1.000 Personenjahre (95 %‐KI), stratifiziert nach AD‐Expositionsstatus und Altersgruppe.

	Inzidenzrate *je 1.000 Personenjahre*	
Altersgruppe (Jahre)	*Exponiert*	*Nicht exponiert*
< 30	2,12 (2,07–2,17)	1,45 (1,44–1,47)
30–40	2,55 (2,49–2,62)	1,64 (1,62–1,67)
40–50	3,04 (2,97–3,11)	2,10 (2,08–2,13)
50–60	5,28 (5,18–5,37)	4,00 (3,96–4,04)
60–70	7,72 (7,60–7,84)	6,01 (5,96–6,06)
70–80	8,92 (8,78–9,07)	7,20 (7,14–7,26)
> 80	10,52 (10,33–10,71)	8,45 (8,36–8,53)

### Sensitivitätsanalysen

**ABBILDUNG 2:**
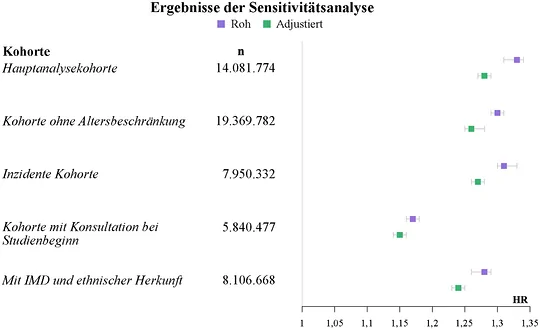
*Forest Plot* mit Zusammenfassung der Sensitivitätsanalyse; gezeigt sind die rohen und adjustierten *Hazard Ratios* (adjustiert für Alter, Geschlecht, Allgemeinpraxis, Asthma, allergische Rhinitis, COPD, koronare Herzkrankheit, arterielle Hypertonie, Herzinsuffizienz, Colitis ulcerosa, Morbus Crohn, Diabetes, Depression, Alkoholabusus, Zigarettenrauchen und Adipositas) und die zugehörigen 95 %‐Konfidenzintervalle für jede Analyse.

Die Ergebnisse der Sensitivitätsanalysen sind in Abbildung 2 zusammengefasst. Die Beschränkung der Kohorte auf inzidente AD (1.382.577 Beobachtungen mit inzidenter AD; aHR: 1,27; 95 %‐KI: 1,26–1,28) und das Entfernen der Altersbeschränkung (aHR: 1,26; 95 %‐KI: 1,25–1,28) führte im Vergleich zur Hauptanalyse sowohl bei dem Modell, in dem nur die Faktoren der Paarungen berücksichtigt wurden, als auch im adjustierten Modell zu etwas geringeren aber insgesamt ähnlichen Effektschätzern (aHR: 1,28; 95 %‐KI: 1,27–1,29).

Die Beschränkung der Kohorte auf Beobachtungen mit einer der früher beschriebenen allgemeinen Aufzeichnungen zum Indexdatum oder innerhalb des vorhergehenden Jahres verringerte die Effektschätzer und somit die aHR auf 1,15 (95 %‐KI: 1,14–1,16). Das Hinzufügen von ethnischer Herkunft und IMD zum adjustierten Cox‐Modell in der Hauptanalysekohorte verringerte die Anzahl der eingeschlossenen Beobachtungen auf 8.106.668 und führte zu einer leichten Verringerung der Effektschätzer (vollständig adjustierte HR: 1,24; 95 %‐KI: 1,23–1,25).

## DISKUSSION

Wir fanden starke Hinweise auf eine Assoziation zwischen AD und HZ mit einem um schätzungsweise 28 % erhöhten Risiko für HZ (95 %‐KI: 27 %–29 %) nach Adjustierung für Alter, Geschlecht, Kalenderzeit, Komorbidität, Zigarettenrauchen, Alkoholabusus und Adipositas. Das Risiko für HZ stieg mit dem AD‐Schweregrad. Die zusätzliche Adjustierung für Exposition gegenüber oralen Kortikosteroiden und konventionellen Immunsuppressiva führte nur zu leichter Verringerung des allgemeinen Effektschätzers. Es gab starke Hinweise auf eine altersbedingte Effektmodifikation, wobei der Effekt von AD auf das HZ‐Risiko bei jüngeren Altersgruppen stärker ausgeprägt war, insbesondere bei Personen unter 50 Jahren.

Im Vergleich zu früheren Studien, in denen die Assoziation zwischen AD und HZ untersucht wurde, fanden wir ein deutlich geringeres zusätzliches Risiko als eine Studie aus Taiwan, in der Versicherungsdaten verwendet wurden,^20^ und eine Studie basierend auf stationären Behandlungen in den USA.^22^ Unsere Ergebnisse stimmen mit denen einer Studie aus dem Vereinigten Königreich von Wan et al.^19^ überein, die eine ähnliche Methode verwendeten. Allerdings lag der Schwerpunkt in dieser Studie nicht spezifisch auf HZ, sondern es wurden allgemein nicht näher bezeichnete VZV‐Infektionen untersucht. Die Studie basierte auf Daten der Primärversorgung des Vereinigten Königreichs aus der THIN‐Datenbank, die insgesamt kleiner und weniger repräsentativ ist als CPRD Aurum.^39^

Adjustierungen für verschiedene Definitionen der Exposition gegenüber oralen Kortikosteroiden und der zeitaktualisierten Verwendung von Immunsuppressiva führten beim univariablen Modell nur zu einer leichten Verringerung der Effektschätzer. Während das mit diesen Behandlungen assoziierte erhöhte Risiko für HZ^40^ in diesen Modellen zum Ausdruck kommt, weisen die Ergebnisse auf ein erhöhtes, von Komorbidität und konventionellen systemischen Behandlungen unabhängiges Grundrisiko für HZ bei Patienten mit AD hin. Da die überwiegende Mehrheit der Beobachtungen vor der Einführung von JAK‐Inhibitoren in die klinische Praxis Ende 2022 aufgezeichnet wurde,^41^ ist der Einfluss dieser neueren Behandlungen auf die Ergebnisse wahrscheinlich zu vernachlässigen. Gleichzeitig weisen die Ergebnisse möglicherweise darauf hin, dass Patienten mit AD besonders anfällig für HZ sind, wenn sie mit JAK‐Inhibitoren behandelt werden.

Wir beobachteten eine beträchtliche Erhöhung des HZ‐Risikos mit steigendem AD‐Schweregrad, insbesondere bei Patienten mit schwerer AD. Da Behandlungen als Surrogat für den Schweregrad der Erkrankung verwendet wurden, ist die Entflechtung der Effekte eines höheren Schweregrads und der behandlungsbedingten Effekte schwierig. Die Ergebnisse der Analysen zu den Auswirkungen oraler Kortikosteroide, potenter topischer Kortikosteroide und systemischer Immunsuppressiva lassen darauf schließen, dass diese Effekte teilweise durch diese Behandlungen vermittelt werden.

Zusätzliche Daten von Krankenhäusern oder Überweisungen von Fachärzten, die leider nicht für diese Studie zur Verfügung standen, wären erforderlich, um die Auswirkung des AD‐Schweregrads auf das HZ‐Risikos genauer zu beurteilen. Der Zusammenhang wird wahrscheinlich sowohl durch den Schweregrad der zugrundeliegenden Erkrankung als auch die behandlungsspezifischen immunmodulatorischen Mechanismen vermittelt.

Die beobachtete Effektmodifikation durch das Alter weist darauf hin, dass die Auswirkung von AD auf das HZ‐Risiko bei jüngeren Erwachsenen stärker ist als bei älteren Patienten. Dies kann auf Faktoren beruhen, die das Grundrisiko beeinflussen, bei der Analyse nicht erfasst wurden und nicht multiplikativ mit AD interagieren.

So könnten Patienten im Alter von über 70 Jahren, die seit 2013 Anspruch auf eine HZ‐Impfung haben,^42^ die HR in dieser Altersgruppe verringert haben, insbesondere wenn sich die Durchimpfungsrate zwischen den exponierten und nicht exponierten Gruppen unterschied. Darüber hinaus könnte ein *Survivorship‐Bias* eine Rolle gespielt haben, da in dieser Studie nur die erste Manifestation von HZ untersucht wurde: Wenn ein relevanter Anteil der Patienten mit AD in einem jüngeren Alter erstmals an HZ erkrankte, wären sie von der Analyse der Gruppen mit höherem Lebensalter ausgeschlossen, mit der möglichen Konsequenz einer Unterschätzung der altersspezifischen HR.

### Stärken und Limitationen

CPRD Aurum ist weitgehend repräsentativ für die Bevölkerung des Vereinigten Königreiches hinsichtlich Geographie, Alter, Geschlecht und sozialer Benachteiligung. Da die Teilnahme von Hausarztpraxen an der Datenbank freiwillig ist und Patienten der Teilnahme widersprechen können, ist die Generalisierbarkeit jedoch möglicherweise beeinträchtigt.

AD ist wahrscheinlich zu einem gewissen Grad unterdiagnostiziert oder unterdokumentiert.^43^ Da der AD‐Status vor dem Studienergebnis erfasst wurde, ist jede Fehlklassifikation des Expositionsstatus wahrscheinlich nicht‐differentiell. Dies würde zu einer Verzerrung der Effektschätzer zur Null führen. Da Personen, die die AD‐Kriterien nicht erfüllten, automatisch als nicht exponiert eingestuft wurden, könnte dies eine Erhebungsverzerrung (*Ascertainment‐Bias*) zur Folge haben.

Patienten mit AD konsultieren ihren Hausarzt aufgrund ihrer Erkrankung wahrscheinlich häufiger. Darüber hinaus enthält die Gruppe der gepaarten Vergleichspersonen möglicherweise Patienten, die ihren Hausarzt nicht oder nur selten konsultierten, während exponierte Patienten ihren Hausarzt mindestens zweimal konsultieren mussten, um die Definition für AD‐zu erfüllen. Dementsprechend könnte eine Konsultations‐Verzerrung die Effektschätzer erhöht haben. Ergebnisse aus der Sensitivitätsanalyse, bei der die Kohorte auf Patienten mit mindestens einer Konsultation im Jahr vor Studienbeginn beschränkt wurde, lassen auf eine gewisse Überschätzung in der Hauptanalyse schließen.

Daten zur Komorbidität wurden den Patientenakten entnommen und wurden daher aufgrund von Unterdiagnosen möglicherweise nicht vollständig erfasst. Darüber hinaus wurden Verschreibungen für Behandlungen nur den Aufzeichnungen zur Primärversorgung entnommen, wobei CPRD die Therapietreue nicht erfasst. Dies limitiert die Sensitivität und Spezifität der Definitionen dieser Variablen.

Wie bereits diskutiert, ist die Unterscheidung zwischen AD‐Schweregrad und Behandlungen aufgrund unserer Methodik begrenzt und sollte mit Vorsicht interpretiert werden. Zukünftige Forschungsarbeiten könnten, sofern verfügbar, alternative, Score‐basierte Klassifikationen des Schweregrads der Erkrankung berücksichtigen, um diese Effekte besser zu entflechten.

Verbleibende *Confounder*‐Effekte durch zielgerichtete AD‐Therapien oder Impfstatus bleiben möglich und sollten in zukünftigen Forschungsarbeiten thematisiert werden. Allerdings ist es unwahrscheinlich, dass die Durchimpfungsrate einen deutlichen Einfluss auf unsere Schätzwerte hatte, sofern sie sich nicht zwischen den Expositionsgruppen unterschied.

In dieser Studie wurde ein dynamisches Kohorten‐Design unter Einbeziehung von sowohl prävalenten als auch inzidenten AD‐Fällen verwendet, um die Stichprobengröße zu maximieren und die Untersuchung langfristiger Effekt zu ermöglichen.^25^ Sensitivitätsanalysen mit Beschränkung auf inzidente AD‐Fälle führten zu ähnlichen Ergebnissen wie die Hauptanalyse. Eine umgekehrte Kausalität ist unwahrscheinlich, da die Studie auf prospektiv erfassten Daten beruhte und Patienten mit HZ‐Diagnose vor dem Indexdatum aus der Analyse ausgeschlossen wurden. Aufgrund der fehlenden Dokumentation hinsichtlich der Gründe für eine Zensur, konnte der Einfluss eines *Loss to Follow‐up* nicht angemessen bewertet werden.

### Schlussfolgerungen

Insgesamt weisen diese Ergebnisse unter Berücksichtigung der verfügbaren Literatur auf ein erhöhtes Grundrisiko für HZ bei Patienten mit AD hin, unabhängig von Komorbidität und konventionellen systemischen Behandlungen. Dieser Effekt ist bei jüngeren Altersgruppen stärker ausgeprägt. Zusammen mit neuen Behandlungsoptionen und der breiteren Umsetzung von HZ‐Impfprogrammen kann die Integration der Hinweise aus dieser Studie in klinische Praxis und Impfrichtlinien den Versorgungsstatus für Patienten mit AD verbessern und die Prävention von HZ unterstützen. Weitere Forschungsarbeiten zu den zugrundeliegenden biologischen Mechanismen sowie Studien, die Daten zu HZ‐Rezidiven, Impfstatus und ein breiteres Behandlungsspektrum einbeziehen, sind angezeigt, um unser Verständnis für diese Assoziation zu verbessern.

## INTERESSENKONFLIKT

Keiner.
